# Dynamic Response of Ti-6Al-2Zr-1Mo-1V Alloy Manufactured by Laser Powder-Bed Fusion

**DOI:** 10.3390/ma17133361

**Published:** 2024-07-08

**Authors:** Hanzhao Qin, Alafate Maierdan, Nan Li, Changshun Wang, Chenglin Li

**Affiliations:** 1 School of Power and Mechanical Engineering, Wuhan University, Wuhan 430072, China; 2021202080029@whu.edu.cn (H.Q.); 2022102080012@whu.edu.cn (C.W.); 2 School of Mechatronic Engineering, Xinjiang Vocational & Technical College of Communications, Urumqi 831401, China; arafat523@126.com; 3 AECC Beijing Institute of Aeronautical Materials, Beijing 100095, China

**Keywords:** laser powder-bed fusion, TA15 alloy, dynamic compression, adiabatic shear bands

## Abstract

Titanium parts fabricated by additive manufacturing, i.e., laser or electron beam-powder bed fusion (L- or EB-PBF), usually exhibit columnar grain structures along the build direction, resulting in both microstructural and mechanical anisotropy. Post-heat treatments are usually used to reduce or eliminate such anisotropy. In this work, Ti-6Al-2Zr-1Mo-1V (TA15) alloy samples were fabricated by L-PBF to investigate the effect of post-heat treatment and load direction on the dynamic response of the samples. Post-heat treatments included single-step annealing at 800 °C (HT) and a hot isotropic press (HIP). The as-built and heat-treated samples were dynamically compressed using a split Hopkinson pressure bar at a strain rate of 3000 s^−1^ along the horizontal and vertical directions paralleled to the load direction. The microstructural observation revealed that the as-built TA15 sample exhibited columnar grains with fine martensite inside. The HT sample exhibited a fine lamellar structure, whereas the HIP sample exhibited a coarse lamellar structure. The dynamic compression results showed that post-heat treatment at 800 °C led to reduced flow stress but enhanced uniform plastic strain and damage absorption work. However, the HIP samples exhibited both higher stress, uniform plastic strain, and damage absorption work owing to the microstructure coarsening. Additionally, the load direction had a subtle influence on the flow stress, indicating the negligible anisotropy of flow stress in the samples. However, there was more significant anisotropy of the uniform plastic strain and damage absorption. The samples had a higher load-bearing capacity when dynamically compressed perpendicular to the build direction.

## 1. Introduction

Additive manufacturing (AM) has revolutionized product design and production across industries such as aerospace, automotive, medical devices, and consumer goods [1]. Unlike subtractive techniques that remove materials, AM builds components layer by layer, enabling unprecedented control over internal architecture [2]. This layer-wise process allows deliberate programming of properties by varying scan patterns, energy density, and thermal history [3,4]. AM thus promises superior or customized properties and improved productivity, expanding the realm of accessible geometries and customized materials [5,6,7].

The Ti-6Al-2Zr-1Mo-1V (TA15) titanium alloy has exceptional strength, creep resistance, corrosion resistance, and bio-compatibility, making it suitable for demanding applications such as aerospace, automotive, chemical, and biomedical sectors [8,9]. For example, the TA15 alloy allows aircraft and ships to withstand stress while reducing weight [6,10]. TA15 components may undergo dynamic forces in such conditions. As a result, impact resistance is a key benchmark when evaluating. In the case of titanium alloys manufactured via AM, investigations into its dynamic compression properties are limited.

Several studies have examined the dynamic compression mechanisms of various titanium alloys. Shi et al. [11] reported that dynamic compression tests on a Ti-8Al-1Mo-1V alloy showed stress fluctuation at early stages due to dislocation multiplication. Work hardening and thermal softening then led to slow hardening. At a strain rate of 2100 s^−1^, adiabatic shear bands (ASBs) began to form. Electron backscatter diffraction analysis found that ultrafine (<1 μm) recrystallized α grains were formed in those shear bands. Xu et al. [12] found that the strength of the Ti-6321 titanium alloy increased significantly with a higher strain rate, displaying strain rate hardening. Twinning dominated the initial plastic deformation mechanism at a low strain range (0.03–0.06). As strain further increased beyond 0.06, the twin density rose before the deformation mechanism transitioned from twinning to dislocation slip. Chen et al. [13] observed that the lamellar α phase in the ASB zone of Ti-5Al-5Mo-5V-1Cr-1Fe recrystallized and re-melted owing to the temperature rise and stress concentration, while the equiaxed α phase aligned into strips along the ASB direction. Twinning transferred between phases, indicating deformation accommodation in the ASBs.

The dynamic compression properties of additive-manufactured titanium alloys under different loading directions are significantly different. Relevant studies have conducted in-depth investigations and discussed the mechanism behind it. Cheng et al. [14] found that the basket-weave microstructure in TC11 alloy fabricated by laser direct energy deposition had much better compression properties than equiaxed ones. Deformation direction relative to α lath orientation determined active slip systems. Loading along <0001> suppressed the activation of the basal/prismatic slip. Loading within the unit triangle enabled slip and caused lath twisting, rotation, and refinement. Lath rotation prompted twinning, which reduced the slip mean free path. ASBs formation led to failure, with α lath thickness reduced below 200 nm within transition zones. Yuan et al. [15] investigated how build orientation affects the compression properties of selective laser-melted Ti-6Al-4V alloy. Vertical (0°), horizontal (90°), and diagonal (45°) samples were tested at strain rates from 0.001 s^−1^ to 4500 s^−1^. Vertical samples had the highest quasi-static yield strength. Under dynamic loading, build orientation strongly influenced yield and flow stress, which increased with strain rate. Adiabatic heating during high-rate compression also positively correlated with strain rate.

A few studies have investigated the dynamic compression properties and deformation mechanisms of TA15 alloys prepared by other additive manufacturing methods. Chen et al. [16] examined how the microstructure and α texture influence the deformation behavior of laser-melted TA15 alloy at high strain rates. Increasing the annealing temperature from 930 °C to 970 °C slightly reduced flow stress by 2% at 3000 ± 100 s^−1^ strain rate. After annealing at 970 °C, the alloy showed a 14.3% higher uniform plastic strain compared to 950 °C annealing, attributed to a significant increase in the volume fraction of the α phase with specific texture, forming {10–12} twins during high strain rate deformation, thereby enhancing plasticity. Xue et al. [17] utilized arc additive manufacturing to produce TA15 samples with 0.1 wt.% boron. They observed an equiaxed pre-β grain structure attributed to controlled arc heat input and boron addition. Heat-treating at 970 °C and 1000 °C led to a noticeable coarsening of the lamellar α phase. The TA15-0.1B samples exhibited low anisotropy at a strain rate of 3000 s^−1^, with the limited impact of heat treatment on the dynamic response. Dynamic compression created {10–12} twin crystals, contributing to a preferred crystallographic orientation along the load direction alongside basal and conical slips.

A comprehensive understanding of the compression behavior of L-PBF TA15 alloys is still lacking. In this study, we systematically investigated the effects of a microstructure and build orientation on the compressive response of L-PBF TA15. Dynamic mechanical testing was conducted on the samples with varied microstructures and load orientations. This study aims to provide new insights into the design and qualification of L-PBF titanium components for demanding applications.

## 2. Materials and Methods

### 2.1. Sample Preparation

**Figure 1:**
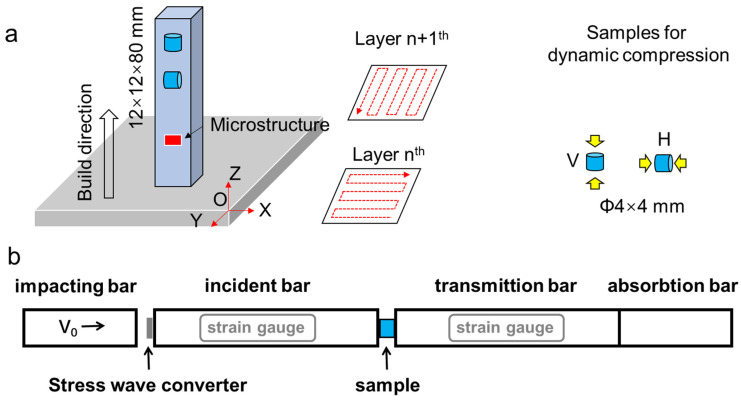
Schematic diagram of the sample fabrication and preparation. (**a**,**b**) Schematic illustration of the spilt Hopkinson pressure bar (SHPB) system.

Gas-atomized TA15 alloy (Ti-6Al-2Zr-1Mo-1V wt%) powders with particle sizes ranging from 10 to 50 μm were used. Additive manufacturing of bulk samples was performed via a commercial selective laser melting system (ASA500M). The following parameters were used: a laser spot diameter of 100 μm, a laser power of 400 W, a layer thickness of 60 μm, a hatch spacing of 120 μm, and a scanning speed of 200 mm/s. A zigzag pattern with a rotation of 90° was used for the printing strategy, as shown in Figure 1a. Samples with a dimension of 12 mm × 12 mm × 80 mm were fabricated. The β-transus temperature of the alloy was measured to be approximately 990 °C using a standard metallurgical method. A post-heat treatment was conducted under 800 °C for 2 h, followed by air cooling. This heat treatment was performed in an air furnace (Tianjin zhonghuan SX-G07123, Tianjin, China). In addition, hot isostatic pressing (HIP) was also performed at a temperature of 930 °C and a pressure of 12 MPa for 3 h, followed by controlled cooling at a rate of 10 °C/min, using a QIH-15 HIP machine (ABB Company, Franklin, TN, USA). Hereafter, the post-heat-treated samples were referred to as HT800 and HIP samples, respectively.

### 2.2. Compression Test

The samples for dynamic compression tests were cut from the block samples and had a dimension of ϕ 4 × 4 mm. The samples of horizontal (H) and vertical (V) directions were prepared to evaluate the mechanical anisotropy. Here, H and V refer to the sample direction in paralleling the loading direction, as shown in Figure 1b. Dynamic compression was conducted in a split Hopkinson pressure bar (SHPB) system, with data recorded by a computer connected to strain gauges, as shown in Figure 1b. The strain rate was fixed at approximately 3000 s^−1^. At least three tests were conducted under each condition to ensure the reliability of the experimental data, and the averages of the results were calculated. Additionally, restraining rings were employed to control the maximum deformation of the samples, thereby investigating the microstructural evolution under different compressive strains. The restraining rings were made of C350 martensitic tempered steel, with a yield strength of up to 1800 MPa and a hardness of 57 HRC, which underwent almost no deformation under the loading conditions. The inner and outer diameters of the restraining rings were 7 mm and 10 mm, respectively. The height of the restraining ring was 3.2 mm, corresponding to a deformation strain of 20%. 

### 2.3. Microstructural Observation

The samples for microstructural characterization were mechanically ground and polished with 5 and 1.5 μm alumina suspensions, finished with a solution of 0.04 μm colloidal silica suspension (80 mL) + H_2_O_2_ (20 mL). Backscattered electron (BSE) imaging and electron backscatter diffraction (EBSD) mapping were used. BSE imaging was performed using an FE-SEM (TESCAN MIRA III, Brno, Czech Republic) at an accelerating voltage of 20 kV and a working distance of 10 mm. EBSD mapping was performed using a field emission gun scanning electron microscope (TESCAN Clara, Brno, Czech Republic) equipped with an AZtec EBSD system at an accelerating voltage of 20 kV. A working distance of 15 mm and a step size of 0.5 μm were used.

## 3. Results

### 3.1. Microstructures

**Figure 2:**
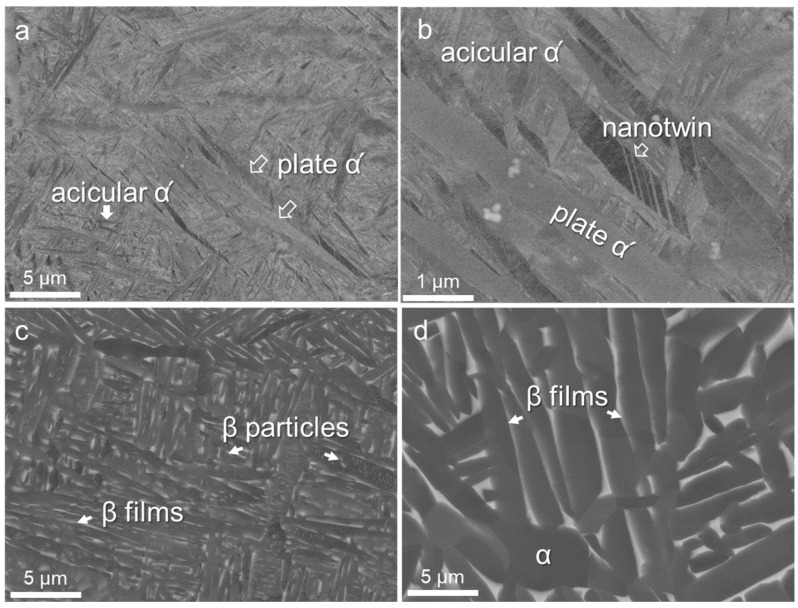
BSE images of the TA15 samples: (**a**,**b**) as-built, (**c**) HT800, and (**d**) HIP.

The microstructures of the as-built and the post-heat-treated samples are shown in Figure 2. It is observed that the as-built sample was mainly composed of lath-like (thickness of about 1 μm, length of 10–50 μm) and needle-like (thickness of about 0.2 μm, length of 2–5 μm) α’ martensite (Figure 2a). Upon annealing at 800 °C, the α’ martensite decomposed into α laths and fine β precipitates or β thin layers (Figure 2b). The α laths derived from the original lath-like and needle-like α’ exhibited distinct length scales: the longer laths transformed from the lath-like α’ (thickness of about 2 μm, length of 10–20 μm), and the shorter laths transformed from the needle-like α’ (thickness of about 0.5 μm, length of 1–4 μm). Furthermore, small β particles formed at the boundaries and within the α laths, with a thin layer of β observed at the lath boundaries (highlighted by the arrows in Figure 2b). The HIP sample exhibited a notably increased thickness compared to HT800, ranging from 2 to 5 μm in thickness and with lengths of 5–20 μm, accompanied by a reduced aspect ratio (Figure 2c). In addition, β primarily existed in the form of thin layers between α laths, with relatively fewer fine β particles presented.

**Figure 3:**
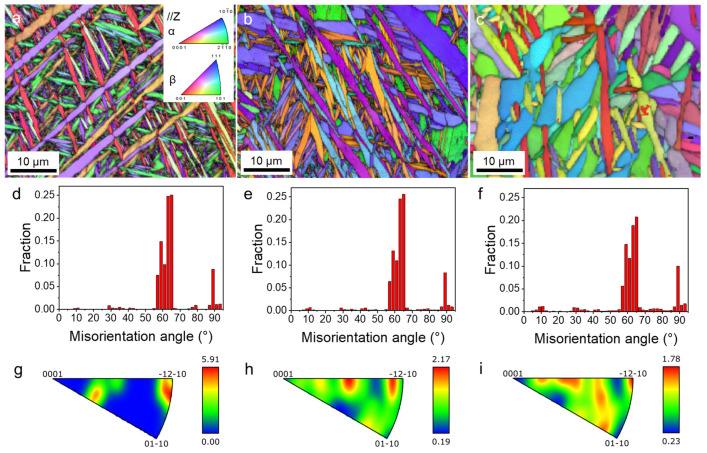
EBSD IPF maps, misorientation maps, and pole figures of the TA15 samples: (**a**,**d**,**g**) as-built, (**b**,**e**,**h**) HT800, and (**c**,**f**,**i**) HIP.

The EBSD maps in Figure 3 show distinct microstructural features in the samples. The as-built sample in Figure 3a showed a heterogeneous microstructure featuring a mix of lath-like and needle-like martensite α’. Upon annealing at 800 °C, the microstructure exhibited coarse α laths, β thin layers, and β particles, as shown in Figure 3b. Notably, the β phase content increased to approximately 3% under this condition. In contrast, the HIP sample exhibited even coarser laths, with a β phase content of approximately 5%. These findings aligned with the BSE observations shown in Figure 2. To quantitatively assess the crystallographic orientation characteristics, we calculated the misorientation distribution under different conditions (Figure 3d–f). Across all the samples, distinct peaks emerged around 60° and 90°, with the 60° peak exhibiting the highest intensity. Notably, the peak intensity at 60° for the HIP sample was notably lower than that of the other two conditions. Moreover, both the HT800 and HIP samples displayed a minor peak around 10°, with HIP exhibiting a higher peak intensity. These peaks likely correspond to the commonly observed Burgers orientation relationship between the β phase and α phase in titanium alloys. Despite these variations, the maximum texture intensity of all the samples remained relatively low, indicating a weak texture. Specifically, the as-built sample exhibited the maximum texture intensity, whereas the HIP sample demonstrated the lowest intensity.

### 3.2. Dynamic Compression Properties

**Figure 4:**
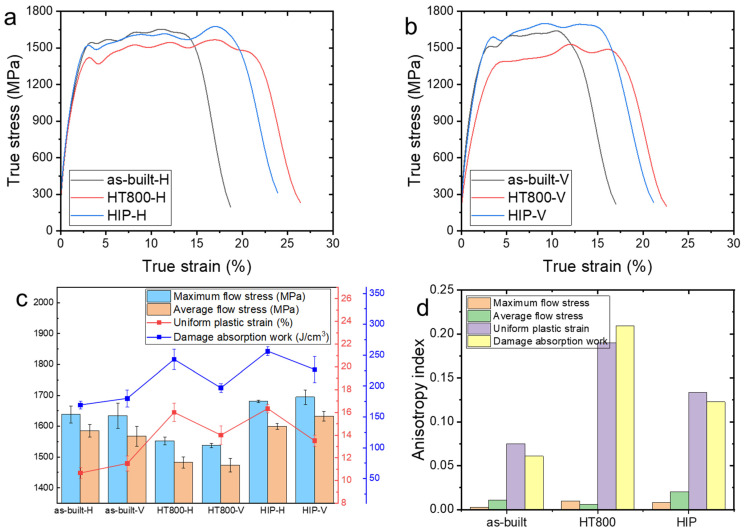
Dynamic compression properties: true stress–strain curves of the H (**a**) and V (**b**) samples, compression properties (**c**), and their anisotropy index (**d**).

Figure 4a,b show the true stress–strain curves of the L-PBF TA15 alloy under dynamic compression with loading along the H and V directions. The stress–strain curve exhibited three distinct stages: elastic, yield, and failure stages. In the elastic stage, all the samples exhibited similar behavior, showing high strength with minimal differences in slope, indicating uniform Young’s modulus in the samples. This uniformity stemmed from the lamellar structure, characterized by a high interface density, requiring significant external force to overcome interface hindrance, especially before plastic deformation. In the yield stage, variations emerged among samples, with stress values fluctuating. This fluctuation might be attributed to the limited deformation time under dynamic compression, impeding heat dissipation and leading to heat accumulation, thereby softening the material. Consequently, dynamic recovery and work hardening mechanisms came into play, driven by dislocation annihilation and rearrangement, resulting in stress fluctuations. As work hardening gradually predominates, stress increases steadily within the fluctuations until reaching peak stress. In the failure stage, stress rapidly decreased, concurrent with the saturation of dislocations and twins within the α laths. Internal shear bands manifested, destabilizing the structure and initiating crack formation. Ultimately, crack propagation and fusion culminated in material fracture failure.

Figure 4c shows the dynamic compression properties of the samples. The average flow stress, uniform plastic strain, and damage absorption work only counted in the yield stage. The as-built samples exhibited a considerably high strength, approximately 1600 MPa. The HT800 samples exhibited a slightly decreased strength, 1500 MPa. The HIP samples exhibited increased strength. The load direction had a very minor effect on the maximum stress and average flow stress in the samples. However, the uniform plastic strain and damage absorption work strongly depended on the load direction. The H samples had higher uniform plastic strain and damage absorption work than the V samples, especially in terms of the HT800 and HIP samples. To quantitatively calculate the mechanical anisotropy in the samples, the following formula was employed:

(1) $$Ix=\left|xH-xV\right|/\bar{x}(0\leq Ix<2)$$

where x represented the stress or uniform plastic strain, and $\bar{x}$ denoted the mean values of the properties in the two directions. The anisotropy of the compression properties of the samples is clearly illustrated in Figure 4d. The dynamic compression samples exhibited no significant anisotropy in terms of maximum stress and average flow stress. However, anisotropies of the uniform plastic strain and damage absorption work were more pronounced, especially in the HT800 and HIP samples. This indicates that the post-heat treatments increased the anisotropy of plastic strain and absorption work.

### 3.3. Fracture Morphology

**Figure 5:**
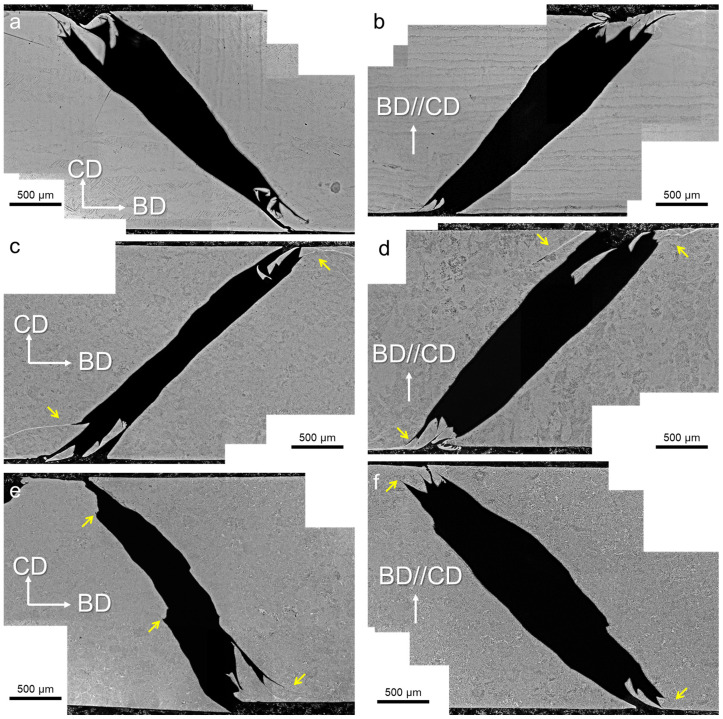
Cross-sectional optical micrograph after dynamic compression: (**a**) as-built-H, (**b**) as-built -V, (**c**) HT800-H, (**d**) HT800-V, (**e**) HIP-H, and (**f**) HIP-V. BD and CD pertain to the build and compress direction.

Figure 5 shows the cross-sectional optical micrographs of the samples after dynamic compression. All the samples exhibited a shear-type fracture, and the cracks aligned approximately 45° to the compression direction. There was no significant difference between the H and V samples, as seen in the macroscopic images. However, the as-built samples exhibited relatively smooth cracks, as shown in Figure 5a,b. The HT800 samples also exhibited relatively smooth cracks, but a few ASBs were observed, as indicated by the arrows in Figure 5c,d. These shear bands demonstrated a clear trend in crack propagation. The HIP samples exhibited serrated cracks, as shown in Figure 5d,e. ASBs could also be observed in these samples, as indicated by the arrows.

**Figure 6:**
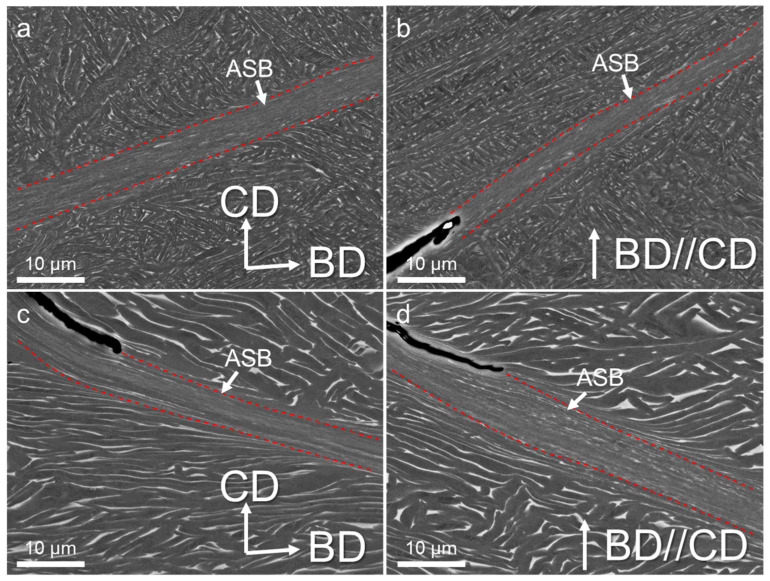
BSE images showing the microstructures of the samples after dynamic compression: (**a**) HT800-H, (**b**) HT800-V, (**c**) HIP-H, and (**d**) HIP-V. BD and CD pertain to the build and compress direction. Red dotted lines indicates the boundries of the ASBs.

SEM observations were performed on the area near the cracks to understand the formation of ASBs, as shown in Figure 6. The ASBs exhibited various widths across the microstructure, showing a diverse range of features within different micro-regions. The microstructure was delineated into three distinct zones: the matrix, the internal domain of the shear bands, and the transition zone. There were notable discrepancy surfaces between the interior of the shear band and the matrix, primarily characterized by exceedingly fine equiaxed grains. This phenomenon arose from the conversion of most plastic deformation into heat energy during dynamic compression, coupled with the brevity of the deformation time, preventing timely heat dissipation. Consequently, a localized temperature surge surpassed the recrystallization threshold, prompting sub-structure rotation and rearrangement, culminating in the formation of dynamic recrystallization grains. The underpinning mechanisms have undergone extensive discussion: Meyers et al. [18] proposed a microstructure evolution model of shear bands grounded in dislocation energy theory. Widely embraced among researchers, the recrystallization theory pivots on dislocation mobility. Under internal stress, dislocations are aligned in a specific configuration. Notably, the defect density within nano-equiaxed grains, predominantly dislocations, largely contrasted with that within the twisted α laths, owing to dislocation annihilation during recrystallization [19,20]. Due to their uniformity and defect-free nature, recrystallized grains stemming from temperature-induced transformations exhibit reduced strength compared to the surrounding deformed grains, thus becoming focal points for localized deformation. In the context of adiabatic shear, cracks tended to manifest more frequently at the periphery of the shear bands. The transition zone presented a juxtaposition of equiaxed and elongated grains, wherein the slab size exerted a pronounced influence on deformation dynamics. Grains within this region contorted in the shear direction, adopting a curved morphology. Adjacent deformed laths aligned nearly parallel to the shear band, resulting in distinctive black-and-white striped patterns delineating the α and β phases. Intense shear strain precipitated the fragmentation of some α and β phases into smaller fragments.

### 3.4. Deformation Mechanism

**Figure 7:**
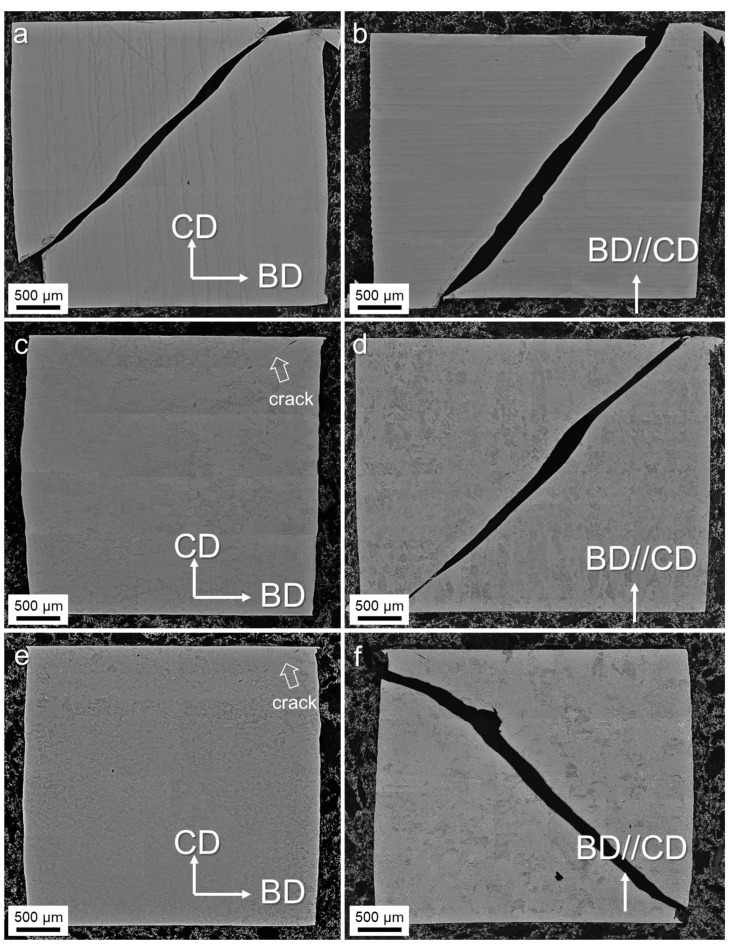
Cross-sectional optical micrographs after being dynamically compressed at 20%: (**a**) as-built-H, (**b**) as-built -V, (**c**) HT800-H, (**d**) HT800-V, (**e**) HIP-H, and (**f**) HIP-V. BD and CD pertain to the build and compress direction.

To further study the deformation mechanism of the alloy samples, the microstructures of the samples subjected to a deformation strain of 20% were investigated, as shown in Figure 7. Complete fracture occurred in the as-built H and V samples, and the HT800-V and HIP-V samples. However, when the load direction was perpendicular to the build direction, only a very small crack occurred in the HT800 and HIP samples, as indicated by the arrows in Figure 7c,d. This indicates that these samples have a higher load capacity when the loading direction is perpendicular to the build direction. In addition, post-heat treatments resulting in coarsening microstructures can also enhance the load capacity.

**Figure 8:**
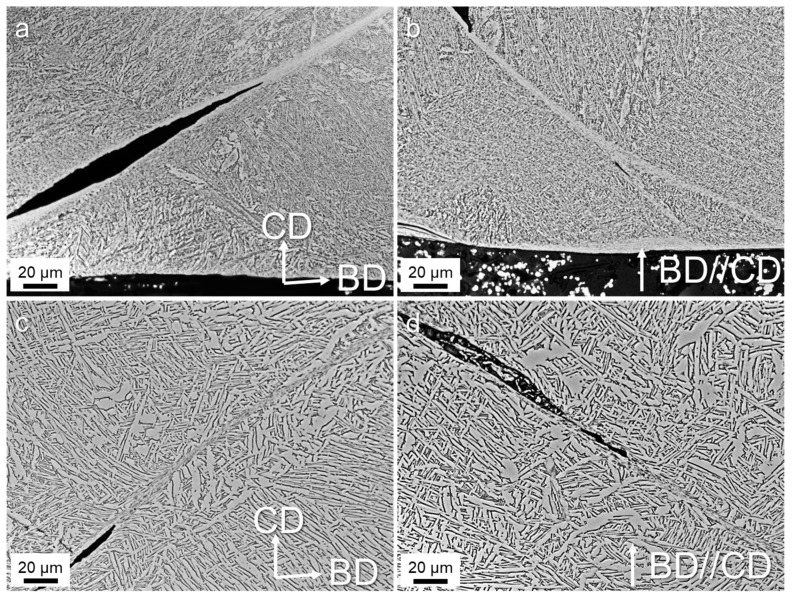
Optical micrographs showing the microstructures of the samples after being dynamically compressed at 20%: (**a**) HT800-H, (**b**) HT800-V, (**c**) HIP-H, and (**d**) HIP-V. BD and CD pertain to the build and compress direction.

**Figure 9:**
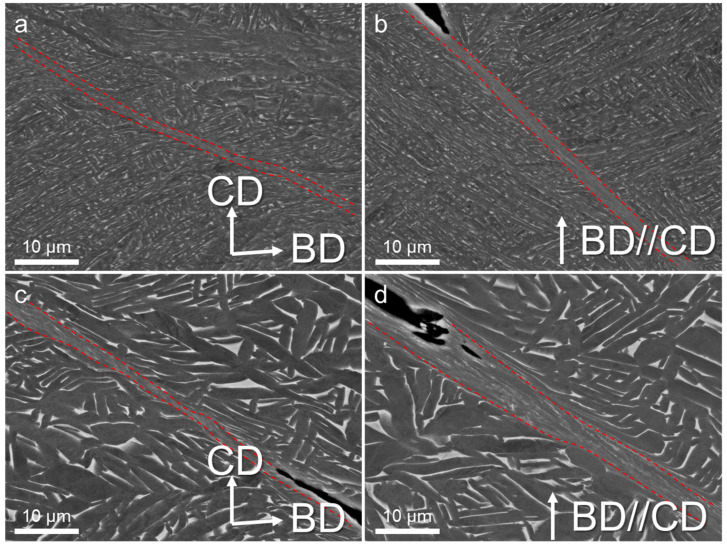
BSE images showing the microstructures of the samples after being dynamically compressed at 20%: (**a**) HT800-H, (**b**) HT800-V, (**c**) HIP-H, and (**d**) HIP-V. BD and CD pertain to the build and compress direction. Red dotted lines indicates the boundries of the ASBs.

Figure 8 and Figure 9 present the ASBs in the samples undergoing dynamic compression deformation at 20%. At this deformation level, micro-cracks emerge within the shear bands, tending to interconnect and expand between adjacent voids and fractures. Xue et al. [21] studied the adiabatic shear behavior of Ti-6Al-4V, attributing the crack initiation to the processes of hole initiation, growth, elongation, rotation, and coalescence within the shear bands. The difference between the shear bands, cracks, and the matrix was clearly illustrated in the HT800 sample. As shown in Figure 8a, the crack grew along the upper edge of the shear band on the left side while it grew along the lower edge on the right side. This suggests that the formation of a large crack was from the coalescence of cracks on either side of the shear band. Similarly, the crack shown in Figure 8b extended toward the center of the shear band, likely to be another possible mechanism of crack formation. Notably, the shear band underwent splitting, with cracks emerging at the split’s outer edge. In contrast, the HIP samples exhibited larger laths, resulting in distinct morphological differences in both cracks and shear bands. Figure 8c shows pronounced distortion in the laths proximate to the crack, with some sections severed by the shear band. Despite the distortion, the remaining laths impeded shear band expansion and crack propagation. Figure 8d shows cracks emerging on either side of the shear band, leading to a complete fracture of the intermediate grains within the band, albeit without further expansion. Coarser laths prove advantageous in restraining shear band growth and crack propagation, which is consistent with the results shown in Figure 4. The relationship delineated among these various shear bands and cracks elucidated the potential failure mechanism of the L-PBF TA15 alloy under dynamic compression.

The BSE images in Figure 9 show that the width of the shear band and the transition zone appeared notably smaller compared to the failure samples in Figure 6. This discrepancy suggests an amplification in the influence range of adiabatic shear with increasing deformation. In contrast to the HT800 samples, the shear bands in the HIP samples exhibited a more irregular pattern. Additionally, the laths within the transition zone displayed evident distortion deformation. The larger laths hindered the uniform expansion and growth of the shear band but extended the transition zone. This phenomenon might elucidate why, despite the HIP samples’ relatively high strength under dynamic compression, the uniform plastic strain did not differ much from that of the HT800 samples.

## 4. Conclusions

In this work, TA15 titanium alloy samples were manufactured through L-PBF. The samples after post-heat treatment at 800 °C and HIP were prepared in order to understand the effect of microstructures on the dynamic deformation behaviors using a split Hopkinson pressure bar at a strain rate of 3000 s^−1^. In addition, the samples were compressed with the load directions parallel and perpendicular to the build direction to understand the anisotropy of the dynamic properties. The microstructural evolution after heat treatment and dynamic deformation were investigated. The main conclusions are as follows:

(1) The as-built sample exhibited a martensitic structure consisting of plate-like and acicular-type α′. The α′ also contained a considerable number of substructures, such as dislocations and nanotwins. Post-heat treatment at 800 °C resulted in the formation of an α + β lamellar structure, consisting of fine α-laths (1–2 μm thick), β particles (nanoscale size distributed in the α-laths), and β films between α-laths. The HIP sample exhibited a coarsened α + β lamellar structure consisting of thick α-laths (2−5 μm thick) and β films.
(2) Post-heat treatment at 800 °C led to reduced flow stress but enhanced uniform plastic strain and damage absorption work. However, the HIP process exhibited both enhanced flow stress, uniform plastic strain, and damage absorption work owing to the microstructure coarsening. The loading direction had little effect on the maximum stress and average flow stress. However, there was a significant anisotropy of uniform plastic strain and damage absorption energy. The HT800 and HIP samples had higher uniform plastic strain and damage absorption energy when they were loaded perpendicular to the build direction. The samples had a higher load-bearing capacity when dynamically loaded perpendicular to the build direction.
(3) The main failure mechanism of the samples was a shear mode, accompanied by the formation of adiabatic shear bands. Cracks were easily initiated and propagated along the shear bands. The coarsened microstructure tended to make the crack propagation difficult.

## Data Availability

The raw data supporting the conclusions of this article will be made available by the authors on request.

## References

[1] Kanishka K., Acherjee B. (2023). Revolutionizing manufacturing: A comprehensive overview of additive manufacturing processes, materials, developments, and challenges. J. Manuf. Process..

[2] Thomas D. (2016). Costs, benefits, and adoption of additive manufacturing: A supply chain perspective. Int. J. Adv. Manuf. Technol..

[3] Li K., Yang T., Gong N., Wu J., Wu X., Zhang D.Z., Murr L.E. (2023). Additive manufacturing of ultra-high strength steels: A review. J. Alloys Compd..

[4] Pothala S. (2023). Recent Advances of Metallic Bio-Materials in Additive Manufacturing in Biomedical Implants—A Review. Mater. Today Proc..

[5] Madigana C.S., Vaddula A., Yerramsetti S.D., Buddaraju K.M. (2023). Additive Manufacturing of Titanium and Nickel-Based Superalloys: A Review. Mater. Today Proc..

[6] Lu K. (2010). The Future of Metals. Science.

[7] Williams J.C., Starke E.A. (2003). Progress in structural materials for aerospace systems11The Golden Jubilee Issue—Selected topics in Materials Science and Engineering: Past, Present and Future, edited by S. Suresh. Acta Mater..

[8] Banerjee D., Williams J.C. (2013). Perspectives on Titanium Science and Technology. Acta Mater..

[9] Gao P.F., Yang H., Fan X.G., Yan S.L. (2012). Microstructural features of TA15 titanium alloy under different temperature routes in isothermal local loading forming. Mater. Sci. Eng. A.

[10] Sun Z., Guo S., Yang H. (2013). Nucleation and growth mechanism of α-lamellae of Ti alloy TA15 cooling from an α + β phase field. Acta Mater..

[11] Shi X., Zhao C., Cao Z., Zhang T., Wang Z., Qiao J. (2019). Mechanical behavior of a near α titanium alloy under dynamic compression: Characterization and modeling. Prog. Nat. Sci. Mater. Int..

[12] Xu X., Ali T., Wang L., Cheng H., Zhou Z., Ning Z., Liu X., Liu A., Zhang B., Cheng X. (2020). Research on dynamic compression properties and deformation mechanism of Ti6321 titanium alloy. J. Mater. Res. Technol..

[13] Chen W., Liu Y., Gang L., Zhou L., Qiu W., Ren Y., Niu Y., Chen J., Li C. (2023). Twinning transfer in a near β-Ti alloy under high strain rate dynamic loading. J. Mater. Res. Technol..

[14] Cheng F., Wang H., Li Z., Cheng X., Zheng D., Zhang S., Hu X., Zhang H., Liu M. (2023). Dynamic compression deformation behavior of laser directed energy deposited α + β duplex titanium alloy with basket-weave morphology. Addit. Manuf..

[15] Yuan Y., Zhang Y., Qiao Y., Xie J., Xu Q., Qi Y., Zhang W., Chen P. (2023). Effect of build orientation on dynamic compressive behaviour of Ti-6Al-4V alloy fabricated by selective laser melting. Mater. Sci. Eng. A.

[16] Chen R., Tan C., You Z., Li Z., Zhang S., Nie Z., Yu X., Zhao X. (2019). Effect of α phase on high-strain rate deformation behavior of laser melting deposited Ti-6.5Al-1Mo-1V-2Zr titanium alloy. Mater. Sci. Eng. A.

[17] Xue L., Xiao J., Nie Z., Hao F., Chen R., Liu C., Yu X., Tan C. (2021). Dynamic response of Ti-6.5Al–1Mo–1V–2Zr-0.1B alloy fabricated by wire arc additive manufacturing. Mater. Sci. Eng. A.

[18] Meyers M.A., Nesterenko V.F., LaSalvia J.C., Xue Q. (2001). Shear localization in dynamic deformation of materials: Microstructural evolution and self-organization. Mater. Sci. Eng. A.

[19] He S., Zeng W., Jia R., Xu J., Zhang X. (2021). The mechanical response and failure mechanism of a near α titanium alloy under high-strain-rate compression at different temperatures. Mater. Sci. Eng. A.

[20] Li Y.S., Tao N.R., Lu K. (2008). Microstructural evolution and nanostructure formation in copper during dynamic plastic deformation at cryogenic temperatures. Acta Mater..

[21] Xue Q., Meyers M.A., Nesterenko V.F. (2002). Self-organization of shear bands in titanium and Ti–6Al–4V alloy. Acta Mater..

